# Molecular surveillance for drug resistance markers in *Plasmodium vivax* isolates from symptomatic and asymptomatic infections at the China–Myanmar border

**DOI:** 10.1186/s12936-020-03354-x

**Published:** 2020-08-05

**Authors:** Yan Zhao, Lin Wang, Myat Thu Soe, Pyae Linn Aung, Haichao Wei, Ziling Liu, Tongyu Ma, Yuanyuan Huang, Lynette J. Menezes, Qinghui Wang, Myat Phone Kyaw, Myat Htut Nyunt, Liwang Cui, Yaming Cao

**Affiliations:** 1 Department of Immunology, College of Basic Medical Sciences, China Medical University, Shenyang, 110122 Liaoning China; 2 Myanmar Health Network Organization, Yangon, Myanmar; 3 Department of Internal Medicine, Morsani College of Medicine, University of South Florida, 3720 Spectrum Boulevard, Suite 304, Tampa, FL 33612 USA; 4 Department of Medical Research, Yangon, Myanmar

**Keywords:** *Plasmodium vivax*, Asymptomatic infection, Symptomatic infection, Drug resistance, Molecular markers, Northeast Myanmar

## Abstract

**Background:**

In the Greater Mekong sub-region, *Plasmodium vivax* has become the predominant species and imposes a major challenge for regional malaria elimination. This study aimed to investigate the variations in genes potentially related to drug resistance in *P. vivax* populations from the China–Myanmar border area. In addition, this study also wanted to determine whether divergence existed between parasite populations associated with asymptomatic and acute infections.

**Methods:**

A total of 66 *P. vivax* isolates were obtained from patients with acute malaria who attended clinics at the Laiza area, Kachin State, Myanmar in 2015. In addition, 102 *P. vivax* isolates associated with asymptomatic infections were identified by screening of volunteers without signs or symptoms from surrounding villages. Slide-positive samples were verified with nested PCR detecting the 18S rRNA gene. Multiclonal infections were further excluded by genotyping at *msp*-*3α* and *msp*-*3β* genes. Parasite DNA from 60 symptomatic cases and 81 asymptomatic infections was used to amplify and sequence genes potentially associated with drug resistance, including *pvmdr1*, *pvcrt*-*o*, *pvdhfr*, *pvdhps*, and *pvk12*.

**Results:**

The *pvmdr1* Y976**F** and F1076**L** mutations were present in 3/113 (2.7%) and 97/113 (85.5%) *P. vivax* isolates, respectively. The K10 insertion in *pvcrt*-*o* gene was found in 28.2% of the parasites. Four mutations in the two antifolate resistance genes reached relatively high levels of prevalence: *pvdhfr* S58**R** (53.4%), S117**N/T** (50.8%), *pvdhps* A383**G** (75.0%), and A553**G** (36.3%). Haplotypes with wild-type *pvmdr1* (976Y/997K/1076F) and quadruple mutations in *pvdhfr* (13I/57**L**/58**R**/61**M**/99H/117**T**/173I) were significantly more prevalent in symptomatic than asymptomatic infections, whereas the *pvmdr1* mutant haplotype 976Y/997K/1076**L** was significantly more prevalent in asymptomatic than symptomatic infections. In addition, quadruple mutations at codons 57, 58, 61 and 117 of *pvdhfr* and double mutations at codons 383 and 553 of *pvdhps* were found both in asymptomatic and symptomatic infections with similar frequencies. No mutations were found in the *pvk12* gene.

**Conclusions:**

Mutations in *pvdhfr* and *pvdhps* were prevalent in both symptomatic and asymptomatic *P. vivax* infections, suggestive of resistance to antifolate drugs. Asymptomatic carriers may act as a silent reservoir sustaining drug-resistant parasite transmission necessitating a rational strategy for malaria elimination in this region.

## Background

*Plasmodium vivax* is the most geographically widespread *Plasmodium* species and also a cause of severe malaria [1–3]. Countries within the Greater Mekong Sub-region (GMS) have endorsed an ambitious plan to eliminate malaria by 2030 [4]. However, the proportion of malaria cases caused by *P. vivax* infection in Myanmar has increased steadily since 2012, especially in border areas [5]. Several features of *P. vivax,* including the formation of hypnozoites, the low density of infection, and the early production of gametocytes favor continuous transmission. *Plasmodium vivax* infections from asymptomatic carriers as a potential silent reservoir for transmission are common in both high- and low-endemic areas of Myanmar [6, 7]. Previous reports of asymptomatic *Plasmodium falciparum* infections carrying genes potentially associated with drug resistance suggest a possible spread of drug-resistant parasites in Myanmar [8, 9]. However, surveys of *P. vivax* drug resistance are scant because most drug resistance studies have focused on *P. falciparum*. Thus, monitoring the emergence and spread of *P. vivax* drug resistance, especially among asymptomatic carriers, is critical to achieve the goal of malaria elimination in the GMS.

Chloroquine (CQ) and primaquine (PQ) combination has been the frontline therapy for treating uncomplicated *P. vivax* cases. *Plasmodium vivax* resistance to CQ was first reported by Papua New Guinea in 1989 [10]. In the GMS, there have been sporadic reports of efficacy studies suggestive of emergence of CQ resistance (CQR) [11–13]. Two recent studies at the China–Myanmar border have demonstrated the declining efficacy of CQ against *P. vivax* and the potential emergence of drug resistance in this parasite [14, 15]. Although sulfadoxine-pyrimethamine (SP) was rarely used to treat *P. vivax*, the substantial selective pressure exerted by the drug is thought to have continued during treatment of *P. vivax* and *P. falciparum* mixed-strain infections, resulting in the emergence of high-grade antifolate resistance in *P. vivax* populations [16]. Molecular surveillance studies indicated that *P. vivax* populations in southwestern Yunnan Province of China bordering Myanmar may be highly resistant to SP [17]. Because artemisinin-based combination therapy (ACT) is also used to treat mixed-species infections [18, 19], *P. vivax* may have been under similar drug selective pressure as *P. falciparum*.

Currently, the molecular mechanisms underlying CQR remain unknown. It has been proposed that *P. vivax* CQR may involve similar molecular mechanisms as in *P. falciparum*. Multidrug resistance 1 gene (*pvmdr1*) and putative transporter protein CG10 gene (*pvcg10* or *pvcrt*-*o*), orthologous to *pfmdr1* and *pfcrt* genes, respectively, have been suggested as possible genetic markers for CQR [20, 21]. However, the first survey of the *pvcrt*-*o* gene in clinical isolates including treatment failure cases failed to identify an association between in vivo CQR with amino acid changes of *pvcrt*-*o*, suggesting the mechanism of CQR in *P. vivax* may be different from that in *P. falciparum* [22]. The K10 insertion in the first exon of *pvcrt*-*o* was the most common but also variable in different parasite populations [20, 23, 24], though it does not appear to correlate with CQR. Analysis of *pvcrt*-*o* mutant isoforms in yeast suggests that at least some *pvcrt* mutations may alter *P. vivax* sensitivity to CQ [25]. Whereas increased expression or copy number of *pvcrt*-*o* was correlated with in vivo CQR in South America [26, 27], such a correlation was not identified in Papua Indonesia, where the level of CQR is high [28]. Recently, using a genetic cross and linkage mapping, upregulated *pvcrt* expression was identified as a mechanism of CQR [29]. In *P. falciparum*, polymorphisms in codons 86, 184, 1034, 1042 and 1246 of the *pfmdr1*gene were reported to be associated with CQR, which correspond to respective positions 91, 189, 1071, 1079 and 1291 in *pvmdr1* [21]. In *pvmdr1*, in vitro studies identified the Y976**F** mutation as a possible marker for CQR in *P. vivax* [20, 30], whereas other studies did not identify such an association [31–34]. Similarly, whereas *pfmdr1* gene amplification was associated with resistance to mefloquine (MQ) in Thailand [30], increased expression of *pvmdr1* and *pvcrt*-*o* was associated with CQR in Brazil [26]. Altogether, the roles of *pvcrt*-*o* and *pvmdr1* in CQR in *P. vivax* are still not resolved [35]. Mutations in *dihydrofolate reductase* (*pvdhfr*) and *dihydropteroate synthase* (*pvdhps*) have been associated with the altered clinical response to SP. F57**L**, S58**R**, T61**M** and S117**N** in *pvdhfr* are linked to pyrimethamine resistance [19, 36–39], while S382**A/C**, A383**G**, and A553**G** in *pvdhps* are responsible for sulfadoxine resistance [40]. Mutations in the propeller region of *P. falciparum kelch 13* (*pfk13*) gene are the main genetic marker for artemisinin resistance [41]. It is logical to determine whether artemisinin drugs have imposed similar selective pressure on the *pfk13* ortholog on chromosome 12 of *P. vivax* (*pvk12)* [42–44].

Drug resistance affects the fitness and virulence of the malaria parasites [45]. This has been demonstrated in *P. falciparum* using in vitro growth competition [46, 47] and inferred from the reversion of resistance-mediating mutations to wild type (WT) in parasite populations after withdrawal of the drug [48]. Since less fit parasites are presumably to produce infections with lower parasitaemia, drug resistance may also affect the clinical presentations of the disease. Some mutations in *pfcrt* and *pfmdr1* were found to have higher prevalence in children with asymptomatic parasitaemia than those with parasitaemia and fever [49]. Similarly, in the GMS, *pfmdr1* amplification was more prevalent in subclinical isolates than clinical isolates [50]. Under the same premise, mutations mediating CQR in *P. vivax* may have differential prevalence in asymptomatic and symptomatic infections.

To test this hypothesis and to obtain more comprehensive information of polymorphisms in candidate drug resistance genes in *P. vivax* in the China–Myanmar border area, *P. vivax* parasites from asymptomatic and acute infections were genotyped at the *pvmdr1*, *pvcrt*-*o*, *pvdhfr*, *pvdhps* and *pvk12* genes.

## Methods

### Study sites and samples

Samples were collected in 2015 in Laiza area of Waingmaw Township, Kachin State, Myanmar, located at the China–Myanmar border. This area has perennial transmission of *P. vivax*, which has become the predominant parasite species, and caused malaria outbreaks in recent years [5, 51]. Passive case surveillance (PCS) was conducted at Laiza hospital and clinics serving the camps of internally displaced people (IDP), where malaria patients presenting with malaria-related signs and symptoms were diagnosed by microscopy and treated. Dried blood spots (DBS) on filter paper containing 200–300 μL of peripheral blood were obtained by a standard finger-prick method, and thick and thin blood smears were prepared to identify parasite species and estimate parasitaemia.

Parasites were identified microscopically by two experienced microscopists. Three seasonal cross-sectional surveys (CSSs) were carried out in March, July and November in two IDP camps and surrounding villages through home visits that involved 5371, 4467 and 3997 participants without any signs or symptoms of malaria, respectively. DBS on filter paper were prepared and stored at − 20 °C for molecular analysis. At the time of the surveys, demographic information was obtained using a structured questionnaire. Parasite density was calculated by quantifying the number of parasites in 500 white blood cells (WBCs) on thick blood smears assuming 8000 WBCs/µL of blood [52]. This study was reviewed by institutional review boards of Pennsylvania State University and China Medical University. Written informed consent/assent was obtained from all malaria patients and participants.

### DNA extraction and molecular identification of *P. vivax* mono-infection

Genomic DNA was extracted from DBS using the QIAamp DNA minikit (Qiagen, Hilden, Germany). *Plasmodium vivax* infection was confirmed by nested polymerase chain reaction (PCR) targeting the 18S ribosomal RNA gene as described previously [53]. Then, the *P. vivax* parasites were genotyped using PCR and restriction fragment length polymorphism (PCR/RFLP) at two polymorphic antigenic markers, *merozoite surface protein*-*3α* (*msp*-*3α*) [54, 55] and *msp*-*3β* [56]. Mixed and multiple infections were excluded, and only *P. vivax* monoclonal infections were used for genotyping drug resistance genes.

### PCR amplification for *pvmdr1*, *pvcrt*-*o*, *pvdhfr*, *pvdhps* and *pvk*12 genes

Fragments of *pvmdr1* (expected amplicon sizes are 604 bp), *pvcrt*-*o* (327 bp), *pvdhfr* (755 bp), *pvdhps* (1259 bp) and *pvk12* (1015 bp), which covered potential drug resistance associated mutations, were amplified by nested PCR using primers reported from previous studies [8, 42, 57, 58] (Additional file 1: Table S1). All reactions were carried out in a total volume of 30 μL containing 19.4 μL water, 0.6 μL of each primer (10 pM), 1.8 μL of MgSO_4_ (25 mM), 0.6 μL of KOD-Plus-Neo DNA polymerase (5 U/μL), 3 μL of dNTP mixture (10 mM each), and 3 μL of 10× PCR buffer following the manufacturer’s instructions (KOD 401, TOYOBO, Japan) with 1.0 μL (5–10 ng) of genomic DNA. The primary and nested PCR conditions for the five genes were the same: initial denaturation at 98 °C for 2 min; 35 cycles of 98 °C for 15 s, 56 °C for 30 s, and 68 °C for 1.5 min; final extension of 68 °C for 5 min. The PCR products were resolved on a 1.2% agarose gel, and the sizes of the PCR products were determined using a 100-bp DNA ladder (3427A, TaKaRa, Japan). PCR products were stored at − 20 °C until sequencing.

### Sequence and statistical analyses

The nested PCR products were purified and sent to SinoGenoMax (Beijing, China) for sequencing using an ABI 3730XL DNA Analyzer. For sequence accuracy, all DNA fragments were sequenced for both strands. Nucleotide and amino acid sequences of *pvmdr1*, *pvcrt*-*o*, *pvdhfr*, *pvdhps* and *pvk*12 were aligned using ClustalW implemented in MEGA7.0.26 with the following reference sequences from the Salvador I strain of *P. vivax*: *pvmdr1* (PVX_080100), *pvcrt*-*o* (PVX_087980), *pvdhfr* (PVX_089950), *pvdhps* (PVX_123230) and *pvk12* (PVX_083080). The single nucleotide polymorphism (SNP) frequency data were analysed using SPSS Statistics 22.0. Fisher’s exact test, Mann–Whitney U test and Student’s t test was used to determine statistical significance (*P* < 0.05). Principal component analysis (PCA) was performed with ClustVis online program (https://biit.cs.ut.ee/clustvis/) on the parasites associated with clinical malaria and asymptomatic infections [59]. The gene sequences reported in this study were deposited in GenBank under accession numbers MT425613–MT425921.

## Results

### Characteristics of study populations

**Fig. 1:**
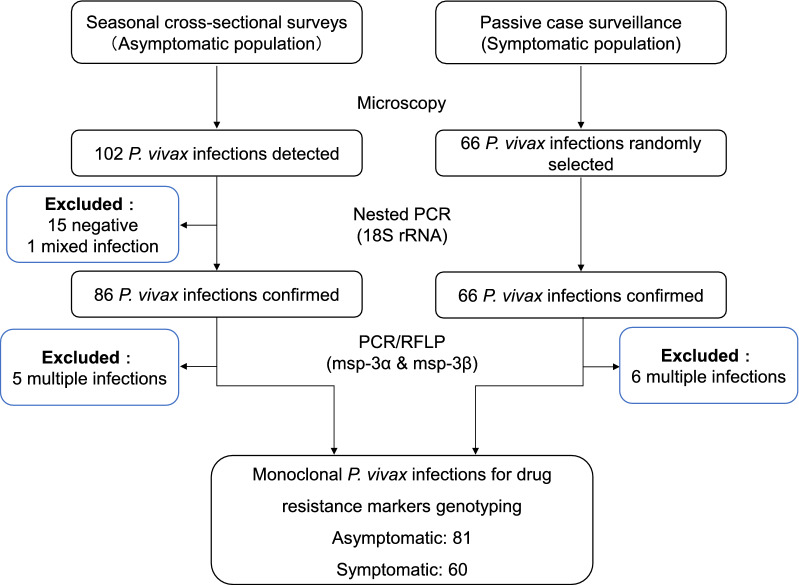
Flow chart of the monoclonal *Plasmodium vivax* isolates screening from asymptomatic and symptomatic populations

To compare *P. vivax* parasites present in asymptomatic and acute infections, CSSs and PCS were conducted in villages/IDP camps and malaria clinics, respectively. Microscopic examination of 13,835 blood smears in three seasonal CSSs identified, 102 asymptomatic *P. vivax* infections, which are defined as individuals with *Plasmodium*-positive blood smears but without any malaria-related symptoms at the time of examination and during the preceding week and the following week (Fig. 1). Nested PCR analysis excluded 15 negative samples and one *P. vivax/P. falciparum* mixed infection. Genotyping by PCR/RFLP of *msp*-*3α* and *msp*-*3β* genes further excluded 5 multiclonal infections, leaving 81 *P. vivax* isolates for genotyping drug resistance markers (Fig. 1). In addition, 66 clinical *P. vivax* infections diagnosed by microscopy were randomly selected from the 2015 PCS samples. After excluding 6 multiple infections, 60 *P. vivax* monoclonal clinical isolates were used for genotyping drug resistance markers (Fig. 1). For the subjects with asymptomatic *P. vivax* infections, males and females were about equally present, and school age children were predominant (Table 1). For patients with patent *P. vivax* infections, males and females were also about equally present, but the age was significantly older than those in the asymptomatic group. For those with acute malaria, about half of them were febrile (axillary temperature ≥ 37.5 °C) at the time of presentation for care, and the majority of them (86.6%) had 1 and 2 days of fever history. As expected, parasite density in symptomatic patients was significantly higher than that in asymptomatic infections (*P* < 0.001, Student’s t test).

**Table 1:** Characteristics of asymptomatic and symptomatic *P. vivax* infections

Characteristics	Asymptomatic	Symptomatic
Number (% male)	81 (53.1)	60 (48.3)
Age [median (IQR)]^a^	14 (11–17)	23 (18–33)***
Fever on day 0 [N (%)]^b^	0	31 (51.7)
Days with fever [N (%)]		
1	0	26 (43.3)
2	0	26 (43.3)
≥ 3	0	8 (13.4)
Parasites density (parasites/µL) [mean (range)]^c^	339 (16–1648)	1485 (64–10,064)***

*IQR* interquartile range

*** Indicates *P* < 0.001

^a^Mann–Whitney U test

^b^Fever is defined as axillary temperature ≥ 37.5 °C

^c^Student’s t test

Eighty-one asymptomatic and 60 symptomatic samples were used for PCR and sequencing analysis of *pvmdr1*, *pvcrt*-*o*, *pvdhfr*, *pvdhps* and *pvk12* genes. The success rates were 57/81 (70.4%) *pvmdr1*, 52/81 (64.2%) *pvcrt*-*o*, 61/81 (75.3%) *pvdhfr,* 41/81 (50.6%) *pvdhps* and 55/81 (67.9%) *pvk12* for asymptomatic samples; and 56/60 (93.3%) *pvmdr1*, 51/60 (85.0%) *pvcrt*-*o,* 55/60 (91.7%) *pvdhfr,* 39/60 (65.0%) *pvdhps* and 55/60 (91.7%) *pvk12* for symptomatic samples.

### *Pvmdr1* and *pvcrt*-*o* genes

Mutations at codons 958, 976, 997 and 1076 in *pvmdr1* were observed in *P. vivax* isolates. The T958**M** mutation was fixed in all parasite isolates and were not considered in analysis. The F1076**L** was present in 97/113 (85.8%) parasites, with 52/57 (91.2%) and 45/56 (80.0%) in asymptomatic and clinical infections, respectively. In contrast, Y976**F** was found only in 3/56 (5.5%) of clinical isolates (Additional file 2: Table S2). Four *pvmdr1* haplotypes were identified, including the WT and three mutants (976**F**, 997**R** and 1076**L**) (Table 2). The haplotype 976Y/997K/1076**L** was the most prevalent (83.2% in total) in both asymptomatic and clinical isolates, with a significantly higher prevalence in asymptomatic than clinical infections (91.2% vs 75.0%, *P *= 0.021). In contrast, the prevalence of the WT was significantly lower in asymptomatic infections than that in clinical infections (1.8% vs 16.1%, *P *= 0.008) (Table 2).

**Table 2:** Prevalence of *pvmdr1*, *pvcrt*-*o*, *pvdhfr* and *pvdhps* haplotypes in asymptomatic and symptomatic infections

Genes (codons)	Haplotypes^a^	# of haplotypes/# of sequenced isolates (%)		
		Asymptomatic	Symptomatic	Total
*pvmdr1* (976/997/1076)^b^	YKF	1/57 (1.8)	9/56 (16.1)**	10/113 (8.8)
	**F**KF	0 (0.0)	3/56 (5.4)	3113 (2.7)
	Y**R**F	4/57 (7.0)	2/56 (3.6)	6/113 (5.3)
	YK**L**	52/57 (91.2)	42/56 (75.0)*	94/113 (83.2)
*pvcrt*-*o* (2/3/K10 insertion)^c^	TI_	37/52 (71.2)	33/51 (64.7)	70/103 (68.0)
	TIK	15/52 (28.8)	14/51 (27.5)	29/103 (28.2)
	T**V_**	0 (0.0)	1/51 (2.0)	1/103 (1.0)
	**I**IK	0 (0.0)	3/51 (5.9)	3/103 (2.9)
*pvdhfr* (13/57/58/61/99/117/173)	IFSTHSI	1/61 (1.6)	0 (0.0)	1/116 (0.9)
	IFST-SI	14/61 (23.0)	7/55 (12.7)	21/116 (18.1)
	IFST**S**SI	13/61 (21.3)	17/55 (30.9)	30/116 (25.9)
	IFST**ST**I	1/61 (1.6)	0/55 (0.0)	1/116 (0.9)
	IF**R**T-**N**I	11/61 (18.0)	9/55 (16.4)	20/116 (17.2)
	I**IRM**HSI	4/61 (6.6)	0 (0.0)	4/116 (3.6)
	I**LRM**H**T**I	0 (0.0)	7/55 (10.9)***	7/116 (6.0)
	I**IRM**H**T**I	17/61 (27.9)	13/55 (23.6)	30/116 (25.8)
	**LIRM**H**T**I	0 (0.0)	2/55 (3.6)	2/116 (1.7)
*pvdhps* (382/383/512/553/571/647)	SAKAEA	13/41 (31.7)	7/39 (17.9)	20/80 (25.0)
	S**G**KAEA	12/41 (29.3)	17/39 (43.6)	29/80 (36.3)
	S**G**K**G**EA	8/41 (19.5)	11/39 (28.2)	19/80 (23.8)
	S**G**KA**Q**A	2/41 (4.9)	0 (0.0)	2/80 (2.5)
	**AG**K**G**EA	3/41 (7.3)	2/39 (5.1)	5/80 (6.3)
	S**GMG**EA	3/41 (7.3)	0 (0.0)	3/80 (3.8)
	S**G**K**G**E**V**	0 (0.0)	1/39 (2.6)	1/80 (1.3)
	**CGEG**EA	0 (0.0)	1/39 (2.6)	1/80 (1.3)

The difference in the major haplotypes between asymptomatic and symptomatic infections was calculated by Fisher’s exact test. * P < 0.05, ** P < 0.01, *** P < 0.001

^a^*Mutant amino acids are shown in boldface*

^b^For *pvmdr1*, the fixed T958**M** was not considered

^c^For *pvcrt*-*o*, _ indicates no **K**10 insertion

More than 30% isolates carried the K10 insertion in the *pvcrt*-*o* gene. Mutations at codons 2 and 3 (T2**I** and I3**V**) were detected in a few clinical isolates but were absent in asymptomatic isolates (Additional file 2: Table S2). WT was the most prevalent haplotype (68.0%), followed by K10 insertion (28.2%) among all the *P. vivax* isolates (Table 2).

### *Pvdhfr* and *pvdhps* genes

For all isolates, mutations in *pvdhfr* at codons 13, 57, 58, 61, 99 and 117 were present in 2/116 (1.7%), 42/116 (36.2%), 62/116 (53.4%), 42/116 (36.2%), 31/116 (26.7%) and 59/116 (50.9%) isolates, respectively. I13**L** and F57**L** were absent in the isolates from asymptomatic populations (Additional file 2: Table S2). The top three mutations in clinical isolates were S58**R** (54.5%), T61**M** (38.2%) and S117**T** (38.2%), while in asymptomatic populations they were S58**R** (52.5%), S117**T** (41.0%) and T61**M** (34.4%), F57**I** (34.4%). Haplotype analysis of *pvdhfr* for all isolates revealed nine distinct allelic forms (Table 2), including the WT haplotype, haplotypes carrying a single mutation (99**S**), double mutations (99**S**/117**T** and 58**R**/117**N**), triple mutations (57**I**/58**R**/61**M**), quadruple mutations (57**L**/58**R**/61**M**/117**T** and 57**I**/58**R**/61**M**/117**T**), and quintuple mutations (13**L**/57**I**/58**R**/61**M**/117**T**). A deletion type (13I/57F/58S/61T/99H-117S/173I) was also identified. The prevalence of the quadruple mutant 57**L**/58**R**/61**M**/117**T** differed significantly in frequency between the asymptomatic and clinical isolates (0% vs 10.9%, *P *= 0.004). Interestingly, the quadruple mutant 57**I**/58**R**/61**M/**117**T** was only observed in isolates from asymptomatic populations (Table 2).

For *pvdhps* gene, eight mutations were identified, including S382**A/C**, A383**G**, K512**M/E**, A553**G**, E571**Q** and A647**V**. Most of the isolates carried A383**G** (75.0%) and A553**G** (36.3%), and they were prevalent in both parasite populations. The K512**M** and E571**Q** mutations were unique in *P. vivax* isolates from asymptomatic populations, whereas S382**C**, K512**E** and A647**V** only presented in clinical isolates (Additional file 2: Table S2). Haplotype analysis of *pvdhps* revealed eight distinct allelic forms (Table 2), including the WT haplotype, and haplotypes carrying a single mutation (383**G**), double mutations (383**G**/553**G** and 383**G**/571**Q**), triple mutations (382**A**/383**G**/553**G**, 383**G**/512**M**/553**G** and 383**G**/553**G**/647**V**), and quadruple mutations (382**C**/383**G**/512**E**/553**G**). Overall, those harbouring a single mutation A383**G** were the most prevalent haplotype, present in 29/80 (36.3%) of clinical isolates. WT at 13/41 (31.7%) was the predominant haplotype in isolates from asymptomatic parasites, followed by the single mutant haplotype with 29.3% frequency (Table 2).

**Fig. 2:**
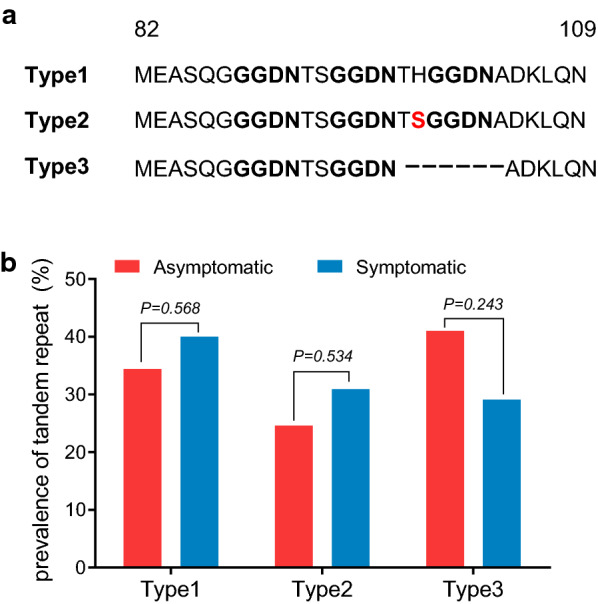
Prevalence of tandem repeat variants of *pvdhfr* in *P. vivax* asymptomatic and acute infections along China–Myanmar border. **a** Sequences alignment of Type 1 (Wild type), Type 2 (H99**S** mutation) and Type 3 (amino-acid repeat regions). Dashes (–) represent tandem repeat deletions. Bold underlined letters indicate the tandem repeat. **b** Prevalence of the three tandem repeat types obtained from asymptomatic and acute *P. vivax* isolates from the China–Myanmar border

Three different tandem repeat variations were found in the *pvdhfr* gene. Type 1 was identical to the Sal I reference sequence, type 2 had a H99**S** mutation, and Type 3 carried a deletion of six amino acids at positions 98–103 (THGGDN) (Fig. 2a). Type 1 accounted for comparable prevalence in asymptomatic (34.0%) and symptomatic (40.0%) infections, respectively. The prevalence of Type 3 in asymptomatic isolates was higher than that in symptomatic infections, and vice versa for Type 2, albeit without statistically significant difference (Fig. 2b). Of note, Type 3 carried the S117**N** mutation rather than S117**T**.

**Fig. 3:**
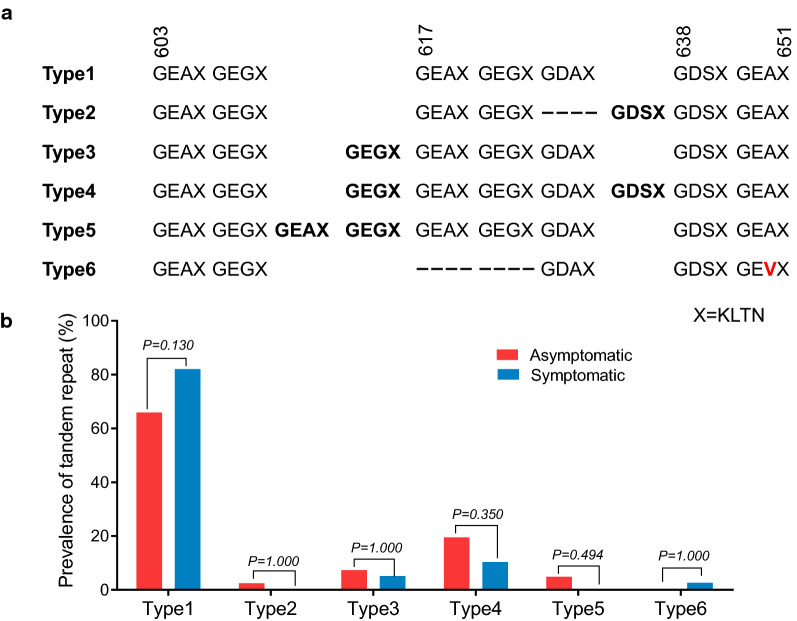
Prevalence of tandem repeat variants of *pvdhps* in *P. vivax* asymptomatic and symptomatic infections along China–Myanmar border. **a** Sequences alignment of Type 1 (wild type) and Type 2–6 (amino-acid repeat regions). Insertions represent tandem repeat at amino acid position 616 and 638. Deletions at amino acid position 617 and 637. Bold letters indicate the tandem repeat. X is a representation of four amino acids (KLTN). **b** Prevalence of six tandem repeat types obtained from asymptomatic and acute infections from the China–Myanmar border area

For *pvdhps* sequences, six different tandem repeat variations were identified with a variable number of tandem repeat unit G (E/D) (A/G/S) KLTN. Type 1, identical to the Sal I reference strain, was the most common in both asymptomatic (65.9%) and clinical (82.1%) infections. Other five types had different deletion or insertion of tandem repeat unit in different amino acids positions (Fig. 3a). Type 3 and Type 4 were found in both types of isolates. For other infrequent variants, Type 2 and Type 5 only presented in asymptomatic populations, while Type 6 was just observed in clinical isolates (Fig. 3b).

### *Pvk12* gene

Sequencing of *pvk12* gene from 110 parasite isolates did not detect any mutations (data not shown).

### PCA

**Fig. 4:**
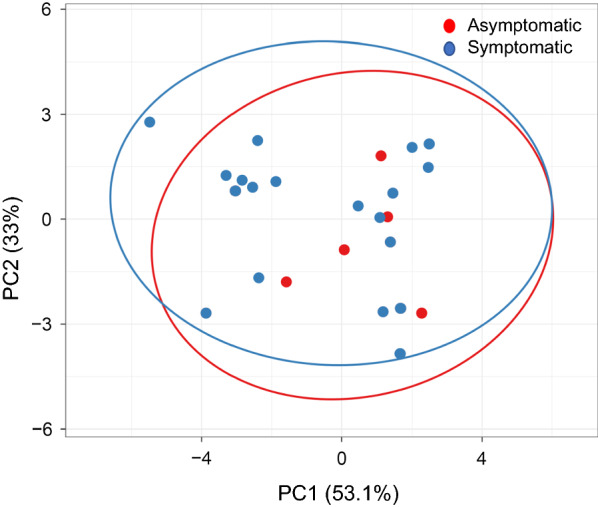
Principal component analysis of *P. vivax* isolates obtained from asymptomatic and symptomatic populations with variables of four drug resistance-related genes (*pvmdr1*, pvcrt-o, *pvdhfr* and *pvdhps*). Twenty-three parasites from each group were used in the analysis. Since some parasites have identical haplotypes, their spots overlapped in the PCA plot. The two types of *P. vivax* population are circled

This study further examined whether parasites populations from the symptomatic and asymptomatic pools were divergent at these potential resistance markers. There were 23 isolates of each population, for which all five genes were successfully sequenced. PCA of *P. vivax* isolates using combined SNPs in *pvmdr1*, *pvcrt*-*o*, *pvdhfr* and *pvdhps* from all 46 samples (Additional file 3: Table S3) showed that parasite populations from asymptomatic and symptomatic parasite populations clustered together (Fig. 4), indicating similarity of the two populations. Analysis of SNPs from each gene separately showed similar results (data not shown 3). It is noteworthy that only five haplotypes were identified from the 23 asymptomatic samples, indicating high prevalence of certain haplotypes.

## Discussion

In Myanmar, CQR in *P. vivax* was reported as early as in the 1990s [12, 13]. Drug resistance in *P. falciparum* and *P. vivax* isolates of asymptomatic malaria carriers has also been reported in high- and low-endemic regions of Myanmar [8, 9]. Recently, along the China–Myanmar border, the therapeutic responses of *P. vivax* malaria to CQ treatment were declining [14, 15]. Thus, this study compared the potential markers for CQR and antifolate resistance in asymptomatic and symptomatic *P. vivax* infections from this region.

The molecular mechanisms underlying CQR are not well understood, but mutations in *pvmdr1* and expression of *pvcrt*-*o* were implicated. For *pvmdr1*, the Y976**F** mutation has been reported in *P. vivax* isolates from many malaria-endemic regions around the world [60–64], and is associated with a decrease in in vitro sensitivity to CQ [20]. In this study, the Y976**F** mutation was relatively rare, with 5.5% detected only in samples from symptomatic infections. This prevalence was much lower than that found in Cambodia (89%) [65] and Thailand (8–25%) [62]. The T985**M** mutation is fixed in all parasite populations in Asia and it is not associated with CQR. Similarly, F1076**L** has not been found to be associated with CQR both in vivo and in vitro drug assays [66]. In this study, F1076**L** reached high prevalence of 85.8%, which was concordant with previous reports from other Asian areas including India, Thailand and Myanmar [23, 60, 61]. The frequency of F1076**L** mutation in asymptomatic infections in Laiza township was twice as much as that in Shwegyin township of Myanmar [8]. The single mutation haplotype 976Y/997K/1076**L** was the most prevalent at the China–Myanmar border, similar to a previous report from India [60], but differed from a report from Yunnan, China, which showed WT as the dominant [67]. These geographical variations in *pvmdr1* gene may suggest different drug selection pressure imposed on *P. vivax* population in these Asian countries.

The role of *pvcrt*-*o* in CQR is controversial. Analysis of *pvcrt*-*o* isoforms in yeast suggest that a single amino acid substitution (S249**P**) slightly increased CQ transport [25], indicating a mild form of CQR. Other studies found that lysine (K) insertion at position 10 of *pvcrt*-*o* gene may be associated with CQR [20, 23]. This study observed a prevalence of 34% of K10 insertion in the *pvcrt*-*o* gene, higher than that found in India (9.4%) and Thailand (0%) [60–62], but lower than that detected in other regions of Myanmar (48.3–72.7%) [23]. A recent study showed correlation of CQR with increased expression of *pvcrt* [29], which could not be evaluated with the DBS samples. Continuous monitoring of clinical efficacy of CQ and candidate molecular markers including *pvcrt*-*o* expression may be necessary to assess CQR in *P. vivax* populations in different parts of the GMS.

Results from this study suggest high-level resistance of the *P. vivax* parasites from the GMS to the antifolate drug SP. Resistance to antifolate drugs in *P. falciparum* and *P. vivax* was found to be associated with point mutations in *dhps* and *dhfr* [68]. For the *pvdhfr* gene, mutations at codons 50, 58, 117 and 173, corresponding to residues 51, 59, 108 and 164 in *pfdhfr*, confer resistance to pyrimethamine [69]. Double mutations (S58**R** and S117**N**) were associated with a high level of resistance in *P. vivax*, whereas quadruple mutations (F57**L**/**I**, S58**R**, T61**M** and S117**T**) were more likely associated with SP treatment failure [38, 70]. Here, the prevalence of double or quadruple mutations (50.7%) was much lower than that found along the Thailand border (100%) and other areas of Myanmar (71%–90%) [23, 61], but much higher than what was found in southern China (9.2%) [71]. Asymptomatic isolates in this study showed a much lower prevalence of both the double and quadruple mutations than that found in southern Myanmar. Compared with the findings reported much earlier in Myanmar and Cambodia where double mutations (S58**R** and S117**N**) accounted for 91.7% to 93.8% of the sequenced samples [36, 72], multiple mutations (more than 2) in the DHFR domain were more frequently in the present study. This may be a warning sign of the growing resistance of *P. vivax* to pyrimethamine over time in Southeast Asia.

Mutations at codons 382, 383, 512, 553 and 585 in *pvdhps*, corresponding to codons 436, 437, 540, 581 and 613 in *pfdhps*, may confer resistance to sulfadoxine. A recent study confirmed that A383**G** was associated with sulfadoxine resistance than other mutations when examined in transgenic rodent parasites expressing PvHPPK-DHPS [73]. In addition, the double mutations A383**G** and A553**G** that possibly cause a disruption in the sulfadoxine-binding site in *P. vivax* were similar to those in *P. falciparum* [40]. At the China–Myanmar border, A383**G** reached 75%. The prevalence of a haplotype with both the A383**G** and A553**G** mutations was 36.5%, obviously lower than at the Thai–Myanmar and–Cambodian borders (61.2%), and in other endemic areas of Myanmar (73.5%) [23, 61].

For *P. falciparum*, triple mutations at codons 51, 59 and 108 of *pfdhfr* and double mutations at codons 437 and 540 of *pfdhps* are associated with SP treatment failures [74]. The combination of *pvdhfr* mutations at codons 57, 58, 61 and 117 and *pvdhps* mutations at codons 383 and 553 was identified in 13 (22%) *P. vivax* isolates, of which 6 (46.2%) were from asymptomatic carriers. These findings suggest that highly resistant *P. vivax* parasites to SP were present among asymptomatic and symptomatic infections at the China–Myanmar border.

Tandem repeats are a unique feature present in the *pvdhfr* and *pvdhps*, but it is not clear whether polymorphisms in these repeat regions contribute to resistance to SP. Tandem repeat region variations were observed in both asymptomatic and symptomatic infections. Consistent with previous studies, parasite isolates based on the *pvdhfr* repeat region were typically separated into three types [67, 75]. Type 1 tandem repeat variant was highly prevalent along with triple, quadruple or quintuple mutations, and about half of Type 3 variant co-existed with the double mutations (58**R**/117**N**). This finding is consistent with that Type 1 and Type 3 are associated with increased resistance to SP [75–78]. It differed from the earlier findings in Cambodia where a large majority of isolates had two GGDN repeat units with double mutations (58**R**/117**N**) [72], indicating that *P. vivax* with antifolate resistance evolved independently in different regions of the GMS. For *pvdhps* gene, five types of tandem repeat variants were identified for the first time in this study. Similar to *pvdhfr*, the majority of *pvdhps* tandem repeat types co-existed with mutations conferring SP resistance. However, further studies are essential to clarify the relationship between these polymorphisms and *P. vivax* sensitivity to SP.

PCA was used to explore if the parasite populations in symptomatic and asymptomatic infections could be differentiated based on the haplotypes of mutations in the five candidate resistance genes. While this method may have limitations to illustrate the genetic relatedness of different parasite isolates, the analysis nonetheless showed that the two clusters largely overlapped. While this supports the notion that asymptomatic infections are important reservoirs for sustaining continued transmission of the parasites, it also showed higher prevalence of certain haplotypes in asymptomatic parasite population. For single-gene haplotypes, the WT *pvmdr1* haplotype was significantly more prevalent in symptomatic patients than asymptomatic carriers, whereas the 976Y/997K/F1076**L** haplotype showed the opposite. In *P. falciparum*, some mutations in the drug transporter genes were found to confer fitness costs [46, 47]. Although the effect of *pvmdr1* mutations on parasite’s fitness is unknown, such differences in the prevalence of the WT and mutant alleles in asymptomatic and symptomatic infections, which had lower and higher parasitaemias, respectively, implies that the F1076**L** mutation may be associated with a fitness loss in the parasites. This mutation varies greatly in different parasite populations [21, 31, 79, 80], and its functional importance for CQR remains to be formally tested. It is also noteworthy that although this study screened more than 13,000 blood samples for asymptomatic infections, only a limited number of slide-positive samples were identified and used in the analysis, thus limiting the sample size and power of the analysis. There were also differences in age distribution between the two groups, which further complicates the comparison as host immunity is correlated with age and exposure. Therefore, although this study provided baseline information on candidate drug resistance genes in *P. vivax* in the China–Myanmar border region, the resistance mechanisms, except for antifolate resistance, demand future investigations.

## Conclusion

All countries in the GMS have set an ultimate goal of eliminating malaria by 2030. One of the main challenges is the resilience of *P. vivax* parasites to control measures, evidenced by the increased proportions of *P. vivax* parasites in many areas of the GMS. Molecular analysis of five potential drug resistance markers in *P. vivax* from the China–Myanmar border area showed prevalence of mutations in *pvmdr1*, *pvdhfr* and *pvdhps*, suggesting resistance to antifolate drugs and possible CQ. The higher prevalence of certain mutant alleles in asymptomatic infections also suggests fitness cost of the mutations, underscoring their potential involvement in drug resistance.

## Supplementary information

**Additional file 1: Table S1.** PCR primer sequences for the amplification of sequences containing *P. vivax pvmdr1, pvcrt*-*o*, *pvdhfr, pvdhps* and *pvk12* genes.

**Additional file 2: Table S2.** Prevalence of *pvmdr1*, *pvcrt*-*o*, *pvdhfr* and *pvdhps* amino acid substitutions in asymptomatic and symptomatic infections.

**Additional file 3: Table S3.** The variables used for each isolate input in the ClustVis online program. Column A indicaes the all variables of each gene. Second row indicates the samples used and Third raw indicates the groups. The reference and alternate allele is shown by 0 and 1, respectively.

## Data Availability

The datasets used and/or analysed during the current study are available from the corresponding authors upon request.

## Abbreviations

ACT: Artemisinin-based combination therapy
CSS: Cross-sectional survey
CQ: Chloroquine
CQR: Chloroquine resistance
CRT: Chloroquine resistance transporter
DBS: Dried blood spot
DHFR: Dihydrofolate reductase
DHPS: Dihydropteroate synthase
GMS: Greater Mekong Sub-region
IDP: Internally displaced people
K12: Kelch-domain protein 12
MDR1: Multidrug resistance 1
MQ: Mefloquine
MSP: Merozoite surface protein
PCR: Polymerase chain reaction
PCR/RFLP: PCR and restriction fragment length polymorphism
PCS: Passive case surveillance
PQ: Primaquine
SP: Sulfadoxine–pyrimethamine
WBC: White blood cell
WT: Wild-type
