# Supplementary material for: Molecular surveillance for drug resistance markers in Plasmodium vivax isolates from symptomatic and asymptomatic infections at the China–Myanmar border
Source: Malar J. 2020 Aug 5;19:281. doi: 10.1186/s12936-020-03354-x (PMC7409419; doi:10.1186/s12936-020-03354-x)
Supplement: Supplementary file 1 — Additional file 1: Table S1. PCR primer sequences for the amplification of sequences containing P. vivax pvmdr1, pvcrt-o, pvdhfr, pvdhps and pvk12 genes. [file 12936_2020_3354_MOESM1_ESM.docx]

Additional file 1: Table S1. PCR primer sequences for the amplification of sequences containing *P. vivax* *pvmdr1, pvcrt-o*, *pvdhfr, pvdhps* and *pvk12* genes.

| Target gene |  | Primer sequence 5'-3' | Product size（bp） | Tm (^o^C) | Reference |
| --- | --- | --- | --- | --- | --- |
| *pvmdr1* | Nested1 | F1 ACGACATGATCCAAACGACA | 2804 | 53 | (Lu et al., 2011) |
|  |  | R1 CTTATATACGCCGTCCTGCAC |  | 58 |  |
|  | Nested2 | F2 GGATAGTCATGCCCCAGGATTG | 604 | 60 |  |
|  |  | R2 CATCAACTTCCCGGCGTAGC |  | 60 |  |
| *pvcrt-o* | Nested1 | F1 ACGGAATCAACCCGAATCCA | 1731 | 60 | (Nyunt et al., 2017) |
|  |  | R1 AGTTTCCCTCTACACCCG |  | 57 |  |
|  | Nested2 | F2 TCCTTGCCGCTGATTCTACG | 327 | 57 |  |
|  |  | R2 GGTAACGTTCATCGGGGGTT |  | 60 |  |
| *pvdhfr* | Nested1 | F1 CACCGCACCAGTTGATTCCT | 983 | 57 | (Ding et al., 2013) |
|  |  | R1 CCTCGGCGTTGTTCTTCT |  | 56 |  |
|  | Nested2 | F2 CCCCACCACATAACGAAG | 755 | 56 |  |
|  |  | R2 CCCCACCTTGCTGTAAACC |  | 60 |  |
| *pvdhps* | Nested1 | F1 AGGAAGCCATTCGCTCAAC | 1438 | 58 | (Nyunt et al., 2017) |
|  |  | R1 GAGATTACCCTAAGGTTGATGTATC |  | 57 |  |
|  | Nested2 | F2 GGTTTATTTGTCGATCCTGTG | 1259 | 54 |  |
|  |  | R2 GAGATTACCCTAAGGTTGATGTATC |  | 57 |  |
| *pvk12* | Nested1 | F1 ATCCAACAGCATTTCCAACT | 2108 | 51 | (Popovici et al., 2015) |
|  |  | R1 CAATTAAAACGGAATGTCCA |  | 50 |  |
|  | Nested2 | F2 ACCACGTGACGAGGGATAAG | 1015 | 57 |  |
|  |  | R2 AAAACGGAATGTCCAAATCG |  | 51 |  |

**Reference**

Ding, S., Ye, R., Zhang, D., Sun, X., Zhou, H., McCutchan, T.F., Pan, W., 2013. Anti-folate combination therapies and their effect on the development of drug resistance in Plasmodium vivax. Scientific reports 3, 1008.

Lu, F., Lim, C.S., Nam, D.H., Kim, K., Lin, K., Kim, T.S., Lee, H.W., Chen, J.H., Wang, Y., Sattabongkot, J., Han, E.T., 2011. Genetic polymorphism in pvmdr1 and pvcrt-o genes in relation to in vitro drug susceptibility of Plasmodium vivax isolates from malaria-endemic countries. Acta tropica 117, 69-75.

Nyunt, M.H., Shein, T., Zaw, N.N., Han, S.S., Muh, F., Lee, S.K., Han, J.H., Thant, K.Z., Han, E.T., Kyaw, M.P., 2017. Molecular Evidence of Drug Resistance in Asymptomatic Malaria Infections, Myanmar, 2015. Emerging infectious diseases 23, 517-520.

Popovici, J., Kao, S., Eal, L., Bin, S., Kim, S., Menard, D., 2015. Reduced polymorphism in the Kelch propeller domain in Plasmodium vivax isolates from Cambodia. Antimicrobial agents and chemotherapy 59, 730-733.
