# Supplementary material for: Molecular surveillance for drug resistance markers in Plasmodium vivax isolates from symptomatic and asymptomatic infections at the China–Myanmar border
Source: Malar J. 2020 Aug 5;19:281. doi: 10.1186/s12936-020-03354-x (PMC7409419; doi:10.1186/s12936-020-03354-x)
Supplement: Supplementary file 2 — Additional file 2: Table S2. Prevalence of pvmdr1, pvcrt-o, pvdhfr and pvdhps amino acid substitutions in asymptomatic and symptomatic infections. [file 12936_2020_3354_MOESM2_ESM.docx]

Additional file 2: Table S2. Prevalence of *pvmdr1*, *pvcrt-o*, *pvdhfr* and *pvdhps* amino acid substitutions in asymptomatic and symptomatic infections.

| Genes | Description ^a^ | # of isolates with amino acid substitutions /# of sequenced isolates (%) | | |
| --- | --- | --- | --- | --- |
|  |  | Asymptomatic | Symptomatic | Total |
| *pvmdr1* | Y976**F** | 0 (0.0) | 3/56 (5.5) | 3/113 (2.7) |
|  | K997**R** | 4/57 (7.0) | 2/56 (3.6) | 6/113 (5.3) |
|  | F1076**L** | 52/57 (91.2) | 45/56 (80.0) | 97/113 (85.8) |
| *pvcrt-o* | T2**I** | 0 (0.0) | 3/51 (5.9) | 3/103 (2.9) |
|  | I3**V** | 0 (0.0) | 1/51 (2.0) | 1/103 (1.0) |
|  | **K**10 insertion | 15/52 (28.8) | 14/51 (27.5) | 29/103 (28.2) |
| *pvdhfr* | I13**L** | 0 (0.0) | 2/55 (3.6) | 2/116 (1.7) |
|  | F57**I** | 21/61 (34.4) | 13/55 (23.6) | 34/116 (29.3) |
|  | F57**L^*^** | 0 (0.0) | 8/55 (14.5) | 8/116 (6.9) |
|  | S58**R** | 32/61 (52.5) | 30/55 (54.5) | 62/116 (53.4) |
|  | T61**M** | 21/61 (34.4) | 21/55 (38.2) | 42/116 (36.2) |
|  | H99**S** | 14/61 (23.0) | 17/55 (30.9) | 31/116 (26.7) |
|  | S117**N** | 18/61 (29.5) | 9/55 (16.4) | 20/116 (17.2) |
|  | S117**T** | 25/61 (41.0) | 21/55 (38.2) | 39/116 (33.6) |
| *pvdhps* | S382**A** | 3/41 (7.3) | 2/39 (5.1) | 5/80 (6.2) |
|  | S382**C** | 0 (0.0) | 1/39 (2.6) | 1/80 (1.3) |
|  | A383**G** | 28/41 (68.3) | 32/39 (82.1) | 60/80 (75.0) |
|  | K512**M** | 3/41 (7.3) | 0 (0.0) | 3/80 (3.8) |
|  | K512**E** | 0 (0.0) | 1/39 (2.6) | 1/80 (1.3) |
|  | A553**G** | 14/41 (34.1) | 15/39 (38.5) | 29/80 (36.3) |
|  | E571**Q** | 2/41 (4.9) | 0 (0.0) | 2/80 (2.5) |
|  | A647**V** | 0 (0.0) | 1/39 (2.6) | 1/80 (1.3) |

^a^ Point mutations are shown in boldface.

^*^ For comparison between asymptomatic carriers and symptomatic patients, only *pvdhfr* F57**L** mutation was statistically significant (*P* = 0.002).
